# SARS-CoV-2 RNA Detection on Disposable Wooden Chopsticks, Hong Kong

**DOI:** 10.3201/eid2609.202135

**Published:** 2020-09

**Authors:** Grace Lui, Christopher K.C. Lai, Zigui Chen, Sylvia L.Y. Tong, Wendy C.S. Ho, Apple C.M. Yeung, Siaw S. Boon, Rita W.Y. Ng, Paul K.S. Chan

**Affiliations:** The Chinese University of Hong Kong Faculty of Medicine, Hong Kong, China

**Keywords:** SARS-CoV-2, transmission, chopsticks, communal dining, Hong Kong, viruses, respiratory infections, coronavirus, 2019 novel coronavirus disease, COVID-19, severe acute respiratory syndrome coronavirus 2

## Abstract

We detected severe acute respiratory syndrome coronavirus 2 (SARS-CoV-2) RNA on disposable wooden chopsticks used by 5 consecutive asymptomatic and postsymptomatic patients admitted for isolation and care at our hospital. Although we did not assess virus viability, our findings may suggest potential for transmission through shared eating utensils.

In late 2019, severe acute respiratory syndrome coronavirus 2 (SARS-CoV-2) emerged in China (*1*), spreading primarily through droplets and contact with respiratory secretions or fecal materials (*2*,*3*). It has been shown that SARS-CoV-2 remains viable on plastic and stainless steel for 72 hours (*4*), and SARS-CoV on wood for 60 hours (*5*). Chopsticks have been essential eating utensils for >3 millennia, particularly in Asia, and are made mainly of wood and plastic; metal chopsticks are found in some countries, such as South Korea. Personal chopsticks are often used to pick food from communal dishes. We investigated whether chopsticks could be a potential vehicle of transmission for SARS-CoV-2.

We recruited 5 consecutive patients admitted for isolation and care at our hospital: 1 patient who was asymptomatic, 2 whose symptoms had subsided, 1 with moderate coronavirus disease (COVID-19) caused by SARS-CoV-2 infection, and 1 with severe COVID-19. Before mealtimes, each patient was given a pair of wooden chopsticks packed in a sealed plastic bag. These chopsticks are widely available in Hong Kong, including in canteens of public hospitals. They are made of plain wood, not bamboo, and not painted with color or lacquer. After mealtimes, we collected the used chopsticks. We dipped the tips of the chopsticks in 1 mL of phosphate-buffered normal saline and shook them for 30 sec to release saliva and oral fluid. We detected SARS-CoV-2 RNA by quantitative reverse transcription PCR (*6*). We collected serial sputum samples and nasopharyngeal and throat swabs to document respiratory shedding and for comparison of viral RNA concentrations among specimen types. The Joint Chinese University of Hong Kong—New Territories East Cluster Research Ethics Committee approved this study.

Patient A, 47-year-old woman, was a close contact of a confirmed case-patient. Her diagnosis was based on a surveillance throat sample collected during quarantine. She was admitted to the hospital for isolation and appeared asymptomatic throughout her stay. A pair of chopsticks collected 2 days after admission (12 days after her last exposure) was positive for SARS-CoV-2 RNA (Figure). Two respiratory samples collected after admission were also positive. High-resolution computed tomography (HRCT) of her lungs revealed small consolidations and ground-glass opacities in both lower lobes, left upper lobe, and right middle lobe.

**Figure:**
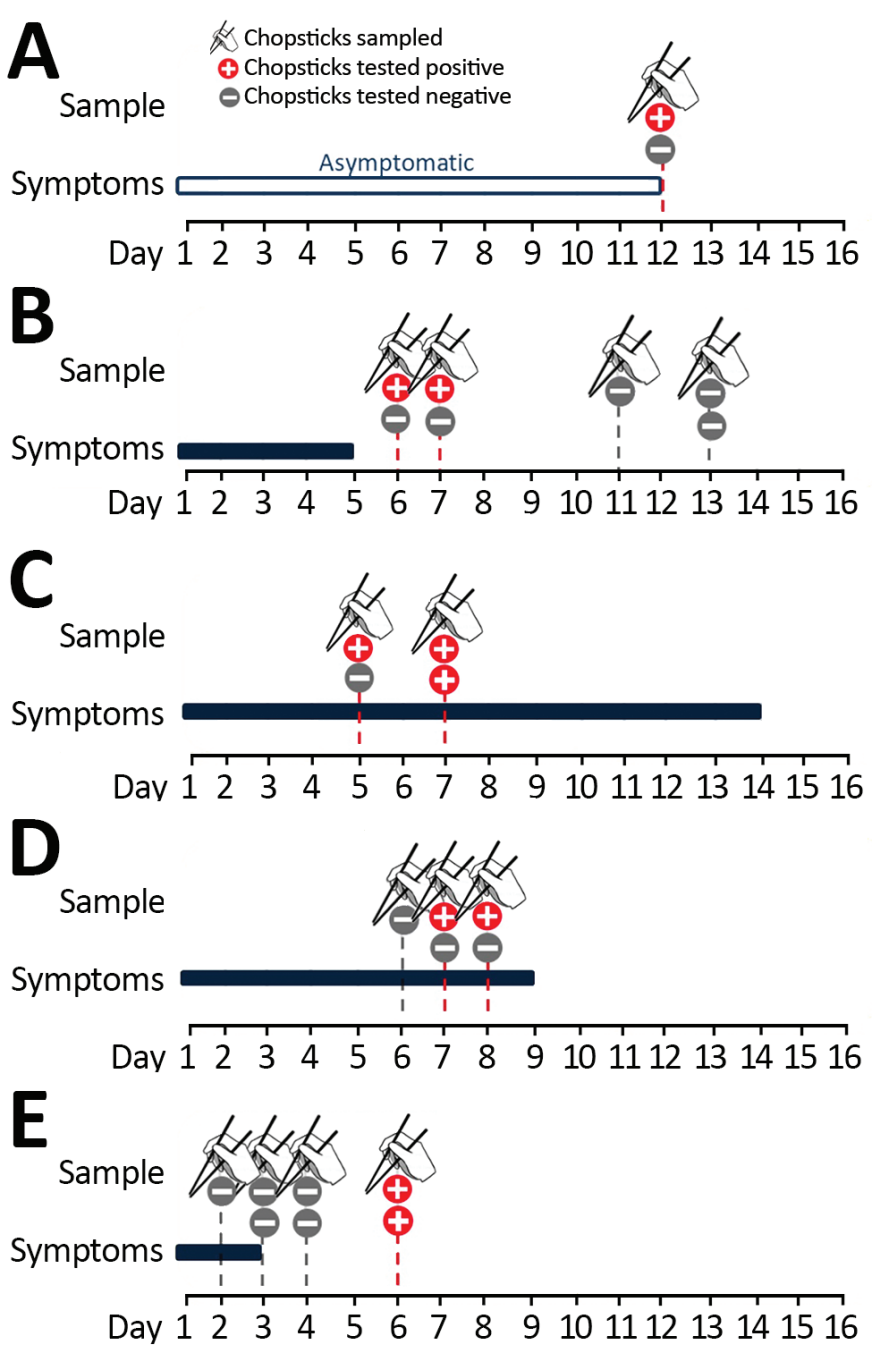
Timelines showing results of severe acute respiratory syndrome coronavirus 2 reverse transcription -PCR testing of chopsticks used by 5 patients in Hong Kong. The results of testing on serial respiratory specimens confirmed that all chopstick samples were collected when patients were shedding viruses from the respiratory tract. A) Patient A was asymptomatic. B) Patient B was postsymptomatic. C) Patient C had severe infection with pneumonia and desaturation. D) Patient D had moderate infection with pneumonia. E) Patient E was postsymptomatic.

Patient B, a 22-year-old woman, had a runny nose, headache, and fever develop on the day she returned from Europe. Her symptoms subsided after admission. Two chopsticks collected 1–2 days after symptoms had subsided were positive for SARS-CoV-2 RNA (Figure). Viral RNA was detected from respiratory specimens until 8 days after symptoms had subsided. HRCT revealed small patchy ground-glass opacity in the anterior segment of the left upper lobe of the lungs. 

Patient C, a 67-year-old man with hypertension and minor coronary artery disease, had fever, cough with whitish sputum, and loose bowel movements develop 2 days after returning from Europe. Chopsticks collected 5 and 7 days after illness onset were positive for SARS-CoV-2 RNA (Figure). All respiratory samples were positive during this period. Chest radiograph revealed right lower lobe infiltrates; the patient’s oxygen saturation fell, requiring supplemental oxygen for 2 days.

Patient D, a 59-year-old man with ankylosing spondylitis and osteoarthritis of both hip joints, had cough with whitish sputum, sore throat, hoarseness, and fever develop. He had no history of travel or contact with known COVID-19 case-patients. Two chopsticks collected 7 and 8 days after illness onset were positive for SARS-CoV-2 (Figure). All respiratory specimens collected during this period were also positive (Appendix Figure). HRCT of the lungs revealed right lower lobe consolidation.

Patient E, the last patient admitted in this study series, was a 26-year-old woman who had fever and headache develop 4 days after returning from Japan; chest radiograph revealed right lower lobe infiltrates. Her fever subsided soon after admission. She developed gastrointestinal upset after receiving lopinavir/ritonavir and was not eating well. Chopsticks collected 1–3 days after symptom onset were negative for SARS-CoV-2, but the 2 pairs collected 3 days after fever had subsided were positive for SARS-CoV-2 RNA (Figure). All respiratory specimens collected from patient E during the study period were positive.

Our study demonstrates frequent contamination of chopsticks with viruses shed from patients with different severity of SARS-CoV-2 infection, including asymptomatic and postsymptomatic patients. In 2 cases, chopsticks were positive after symptoms had subsided. Our main limitations were the small sample size and no attempt to investigate virus viability. Nevertheless, the possibility of chopsticks or other dining utensils as a vehicle for transmission of this novel coronavirus should not be ignored. Although restaurants often provide extra serving chopsticks for picking food from shared dishes, in practice it is easy to mix up personal and serving chopsticks during a meal. Furthermore, serving chopsticks are not commonly used when dining with family and close friends. Restrictions on communal meals should be implemented as part of social distancing strategies, especially in communities with a custom to share dishes. Chopsticks and other eating utensils used by patients should be handled and disposed of as infectious substances as a standard infection control practice.

Appendix Additional information about SARS-CoV-2 RNA detection on chopsticks, Hong Kong.
