# Supplementary material for: SARS-CoV-2 RNA Detection on Disposable Wooden Chopsticks, Hong Kong
Source: Emerg Infect Dis. 2020 Sep;26(9):2274–6. doi: 10.3201/eid2609.202135 (PMC7454084; doi:10.3201/eid2609.202135)
Supplement: Appendix — Additional information about SARS-CoV-2 RNA detection on chopsticks, Hong Kong. [file 20-2135-Techapp-s1.pdf]

Article DOI: https://doi.org/10.3201/eid2609.202135

SARS-CoV-2 RNA Detection on Disposable Wooden Chopsticks, Hong Kong

Appendix

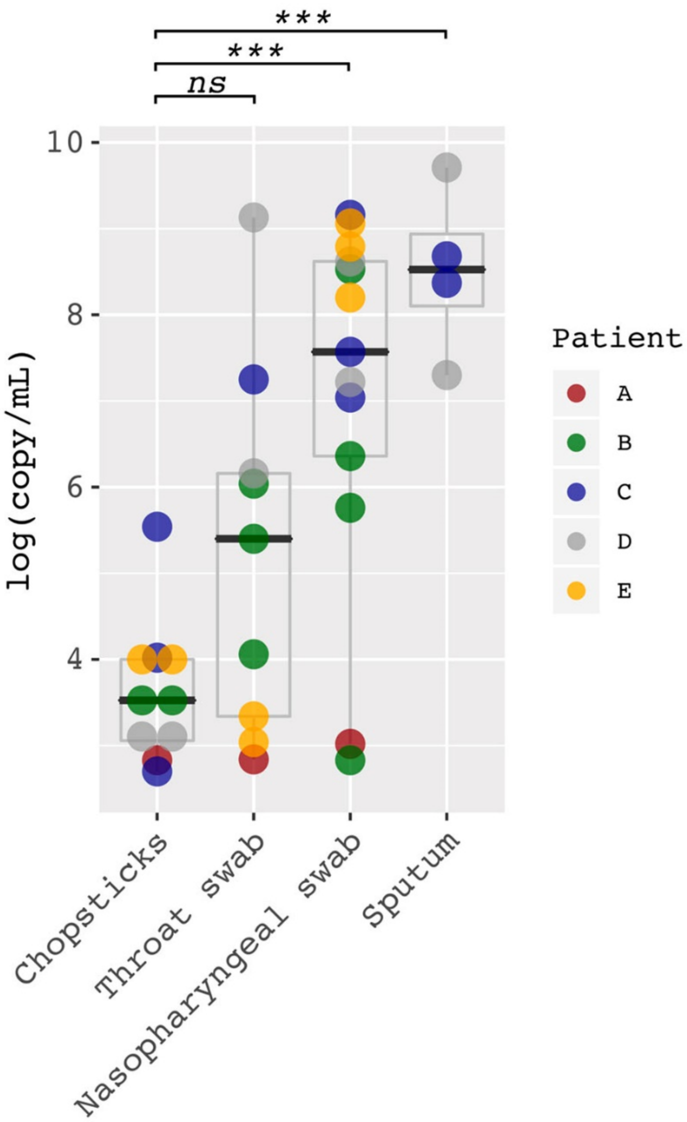

Appendix Figure. Concentration of SARS-CoV RNA detected from chopsticks and respiratory specimens. The cycle threshold (Ct) values of real-time RT-PCR were converted into viral RNA copies based on a standard curve prepared from 10-fold serial dilutions of know copies of plasmid containing the full N gene (2019-nCoV_N_Positive Control, Integrated DNA Technologies, https://www.idtdna.com).

***The concentration of SARS-CoV-2 RNA detected from chopsticks (median 3.4×10^3, interquartile range [IQR] $1.0–10.0 \times 10^3$) viral copies/mL was significantly lower than those of nasopharyngeal swabs ($3.68 [0.23–42.04] \times 10^7$), and sputum samples ($3.54 [1.27–27.92] \times 10^8$), $p<0.001$ by *t*-test for both comparisons; ns, no significant difference in viral copy levels between chopsticks (median [IQR]: $3.4 [1.0–10.0] \times 10^3$) and throat swab samples ($2.50 [0.22–14.4] \times 10^5$ viral copies/mL, $p = 0.058$ by *t*-test). Patient A, who was asymptomatic, had very low viral levels in chopsticks and respiratory samples (range $0.7–1.0 \times 10^3$ viral copies/mL).
